# Survival benefits of neoadjuvant chemo(radio)therapy versus surgery first in patients with resectable or borderline resectable pancreatic cancer: a systematic review and meta-analysis

**DOI:** 10.1186/s12957-019-1767-5

**Published:** 2019-12-31

**Authors:** Long Pan, Jing Fang, Chenhao Tong, Mingyu Chen, Bin Zhang, Sarun Juengpanich, Yifan Wang, Xiujun Cai

**Affiliations:** 1 Key Laboratory of Laparoscopic Technique Research of Zhejiang Province, Department of General Surgery, Sir Run Run Shaw Hospital, Zhejiang University School of Medicine, Hangzhou, 310016 China; 2 Zhejiang Province Medical Research Center of Minimally Invasive Diagnosis and Treatment of Abdominal Diseases, Hangzhou, 310016 China; 3 Institute of Minimally Invasive Surgery of Zhejiang University, Hangzhou, 310016 China; 4 Department of General Surgery, Shaoxing People’s Hospital, Zhejiang University School of Medicine, Shaoxing, 312000 China

**Keywords:** Pancreatic adenocarcinoma, Neoadjuvant chemo(radio)therapy, Surgery first, Survival benefit, Meta-analysis

## Abstract

**Background:**

Pancreatic adenocarcinoma is a highly lethal malignancy. Neoadjuvant chemo(radio)therapy [NAC(R)T] is recommended to use for borderline resectable pancreatic cancer (BRPC) and high-risk resectable pancreatic cancer (RPC), but no high-level evidence exists.

**Methods:**

We searched PubMed, EMBASE, Web of Science, and Cochrane library to identify trials comparing survival data of NAC(R)T with SF for RPC or BRPC. Overall survival (OS) was synthesized in analysis of all the patients (intention-to-treat [ITT] analysis) and resected patients respectively.

**Results:**

The meta-analysis included 17 trials with 2286 participants. For BRPC, NAC(R)T improved OS both in ITT analysis (HR, 0.49; 95% CI, 0.37–0.65; *P* < 0.001) and in analysis of resected patients (HR, 0.66; 95% CI, 0.51–0.85; *P* = 0.001) in comparison to SF, accompanied with comparable overall resection rate [odds ratio (OR), 0.69; 95% Cl, 0.41–1.16; *P* = 0.159]. Disease-free survival, R0 rate, and recurrence were also in favor of NAC(R)T. For RPC, OS in analysis of resected patients was higher with NAC(R)T (HR, 0.75; 95% CI, 0.63–0.89; *P* = 0.001), but OS in ITT analysis was similar (HR, 1.02; 95% CI, 0.85–1.22; *P* = 0.818). The overall resection rate (OR, 0.50; 95% Cl, 0.25–0.99; *P* = 0.048) was lower, but R0 rate was higher with NAC(R)T. No differences in disease-free survival and recurrence between NAC(R)T and SF. Survival benefits of NAC(R)T basically persisted across sensitivity and subgroup analyses.

**Conclusions:**

This meta-analysis demonstrates that NAC(R)T can provide survival benefits in BRPC patients and a subgroup of RPC patients compared with SF. Future research should focus on investigating the potential biomarkers to screen the subgroup of RPC patients who can benefit from neoadjuvant therapy.

**Trial registration:**

CRD42018103086.

## Introduction

Pancreatic ductal adenocarcinoma (PDAC) portends an overall poor prognosis and is expected to become the second lethal malignancy in the USA by 2030 [1, 2]. Although surgery remains the only curative-intent treatment for PDAC, the management based on surgery first (SF) has not substantially improved the survival of patients with potentially resectable disease over the past two decades, even after the effort of adjuvant therapy (AT) [2–4]. The main reason is the early recurrence caused by micrometastases that were not undetected before surgery [3, 5, 6]. Based on these clinical evidence together with other preclinical evidence, PDAC even in early stage, analogous to breast cancer, should be recognized as a systemic disease [2, 7, 8]. Recently, neoadjuvant chemo(radio)therapy [NAC(R)T] is proposed as a new therapeutic strategy for early systemic treatment to increase completeness of resection (R0 rate) and control systemic micrometastases [3, 9]. The newest National Comprehensive Cancer Network (NCCN) guidelines, version 2.2018, recommended NACRT for the management of borderline resectable pancreatic cancer (BRPC). Also, NACRT is considered to be used in high-risk resectable pancreatic cancer (PRC). However, the recommendation of NCCN guidelines lacks high quality evidence [10, 11]. It is controversial for the application of NAC(R)T to RPC or BRPC in the real world, particularly in RPC, which is still intensely discussed at the European Society for Medical Oncology (EMSO) World Congress on Gastrointestinal Cancer 2019. Although there are several randomized controlled trials (RCTs) indicating NACRT increases survival in resectable or borderline resectable PDAC, the trials are limited by small sample sizes [9, 12]. It is still necessary to pool the existing studies to perform a meta-analysis. Indeed, some scholars have done relevant meta-analyses, but most of them are single-arm meta-analyses, such as a recent meta-analysis by Versteijne et al. that lack direct comparison and ignore interstudy heterogeneity [11, 13, 14]. Other published meta-analyses did not focus on survival benefits [15]. Additionally, it is a fact that the definition of RPC and BRPC has undergone several changes over time, which leads to the existence of mixture of RPC and BRPC in the population of included studies according to current standard of resectability status. From this point of view, interstudy heterogeneity exists in all previous meta-analyses.

Hence, we only included comparative studies and reclassified the population as RPC, RPC/BRPC, and BRPC in each study on a basis of the criteria of resectability status in the NCCN guidelines version 2.2018 and conducted this meta-analysis to compare survival benefits of neoadjuvant chemotherapy with or without radiotherapy [NAC(R)T] to SF with or without AT for patients with RPC or BRPC.

## Material and methods

This meta-analysis was performed according to the Preferred Reporting Items for Systematic Reviews and Meta-Analyses (PRISMA) statement [16]. The protocol for this meta-analysis is registered at PROSPERO (CRD42018103086).

### Search strategy

A systematic literature search of online database including PubMed, Web of Science, EMBASE, and Cochrane library was performed for published articles from the inception dates to January 10, 2019. The combination of heading terms and keywords were used to search comprehensively and precisely. The relative terms were as follows: “pancreatic neoplasms,” “surgery,” “resection,” and “neoadjuvant.” The language of articles is limited to English. A detail description of the search is available in Additional file 1: Table S1. Besides, we also reviewed the references of included studies to identify additional literatures.

### Study selection, data extraction, and quality assessment

Two independent investigators (L.P., J.F.) screened articles according to the inclusion and exclusion criteria (Additional file 1: Table S2). The same two researchers independently extracted data and evaluated methodological quality of articles, using a Microsoft excel database to record all available data. For quality assessment, RCTs and non-randomized comparative trials (NRCTs) were respectively evaluated by utilizing the Cochrane risk of bias and the modified Methodological Index for Non-randomized Studies (MINORS) score (Additional file 1: Table S3) [17, 18]. Any disagreement was resolved by another investigator (Y.F.W.).

### Study definition and outcomes of interest

The definition of “borderline resectable” has changed over time and varies in the published literature. In present study, we use the definition of RPC and BRPC in the NCCN guidelines version 2.2018 (Additional file 1: Table S4) to reclassify the study population in included trials as RPC, BRPC, and RPC/BRPC based on the detailed description in the included articles. In RPC/BRPC, the study population in trials mixed with RPC patients and BRPC patients. Stratified analysis (RPC + BRPC, RPC, BRPC) was performed, and RPC + BRPC group contains RPC patients, BRPC patients, and RPC/BRPC patients. The resectability status of PDAC in each of the articles included is discussed and confirmed by all the authors.

The primary outcomes were OS. The hazard ratio (HR) with 95% confidence intervals (CIs) for OS were obtained directly based on data from multivariate Cox proportional hazards regression models in the included literatures. If studies did not offer HRs and 95% CIs, the method provided by Tierney et al. was used to calculate HRs from Kaplan-Meier curves [19]. The second outcomes include 1-, 3-, and 5-year survival rates (1-, 3-, and 5-YSRs), disease-free survival (DFS), recurrence rate, overall resection rate, R0 rate, and pathological positive lymph node (pN+) ratio. The 1-, 3-, and 5-YSRs were acquired from Kaplan-Meier curves, if the studies did not present these data.

### Statistical analysis

HRs and 95% CIs were estimated for OS and DFS using an inverse variance model to pool the data. The pooled odds ratios (ORs) with 95% CIs were estimated for dichotomous outcomes. Between-study heterogeneity was calculated using Higgins’ *I*^2^ and *I*^2^ > 50% indicated significant heterogeneity [20]. A random-effects model was used to pool data when *I*^2^ > 50%, while a fixed-effects model was chosen when *I*^2^ < 50% [21]. The 1-, 3-, and 5-YSRs for NAC(R)T and SF were calculated by single-arm meta-analysis and were presented graphically using bubble plots. Various sensitivity analyses were conducted to observe the stability of results along with extraction of matched baseline characteristics of included trials: (1) matched patient factors, (2) matched tumor size, (3) matched vascular resection, (4) matched initial CA19-9 level, (5) matched tumor factors, (6) matched patient and tumor factors, (7) pancreatic head cancer (≥ 80% of patients), (8) matched AT, and (9) Asians. *χ*^2^ tests and independent *t* tests were respectively used to identify matched baseline factors for dichotomous and continuous variables, if included studies did not provided relevant *P* value. To assess the effects of covariates on the pooled estimates, subgroup analysis and meta-regression analysis were conducted respectively. Publication bias was detected using funnel plots, Begg’s tests, and Egger’s tests [22]. Two-sided *P* < 0.05 was considered as statistical significance. All statistical analyses were performed using STATA/SE version 15.0 (StataCorp LP, College Station, TX).

## Results

### Study selection and quality assessment

**Fig. 1:**
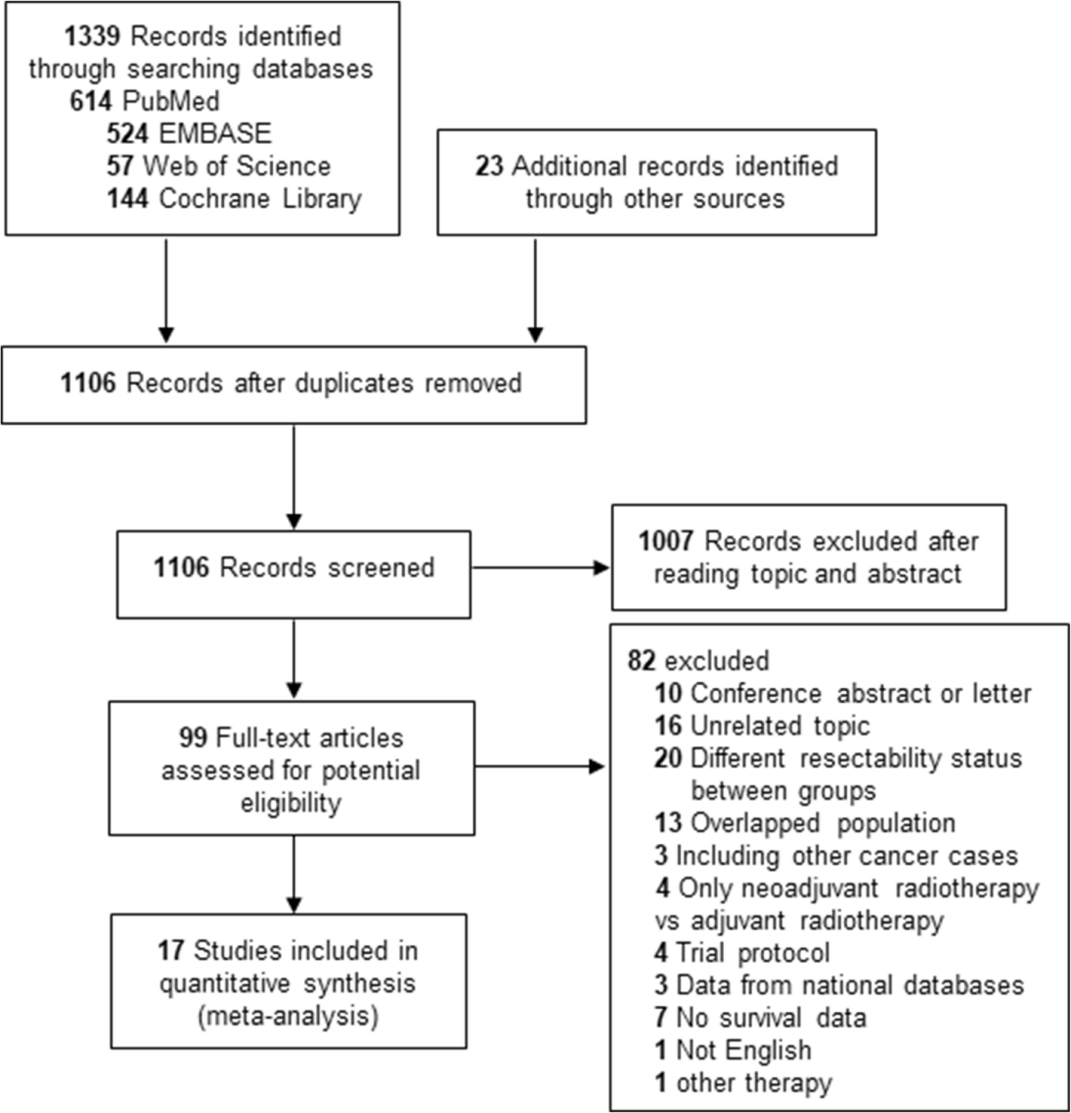
Trial selection process

A total of 1362 records were obtained, of which 99 records were screened fully. Finally, 17 studies consisting of 21 data sets were included with 2286 participants (Fig. 1) [3, 9, 12, 23–36]. Three studies whose data were derived from Surveillance, Epidemiology, and End Results database or National Cancer Database were excluded because these studies had overlapped study population with those studies from individual hospitals [10, 37, 38]. Nine studies [3, 12, 23, 24, 27, 30, 31, 34, 36] included RPC patients and seven studies [3, 9, 26, 31, 32, 34, 35] included BRPC patients. The baseline characteristics, quality score, and matched factors (sex, age, tumor size, tumor size, CA19-9, vascular resection, AT) in each studies included are summarized in Tables 1 and 2. All the studies except Jiang et al. [27] used at least chemotherapy as neoadjuvant reagents. In the study by Jiang et al., 28% of patients only received neoadjuvant radiotherapy without chemotherapy. A sensitivity analysis had been performed by removing Jiang et al. in this meta-analysis. All retrospective trials achieved 12–15 points according to MINORS scores with a total of 16 points. Detailed results of quality evaluation of the RCT and NRCTs are shown in Additional file 1: Table S5 and S6.

**Table 1:** Characteristics and quality assessment of the included studies

Study, year, country	Study type, period	Resectability status	Definition of status	Neoadjuvant treatment (proportion + protocol)	Quality score
Barbier et al. [23], 2011, France	Retro, 1997–2006	RPC	Tumor surrounding ≤ 180° of the circumference of SMV/PV, no tumor contact to CA and SMA, and no occlusion of SMV/PV confluence.	Chemo: 100%, 5-FU + cisplatine Radio: 100%, 45 Gy	14
Papalezova et al. [24], 2012, America	Retro, 1999–2007	RPC	No evidence of tumor extension to SMA, CA, CHA, SMV, and PV. Radiographically borderline resectable or unresectable disease was excluded.	Chemo: 100%, capecitabine or infusional 5-FU Radio: 100%, 45 or 50.4 Gy	13
Tajima et al. [25], 2012, Japan	Retro, 2006–2009	RPC/BRPC	No detailed statement, but potentially resectable diseases were included.	Chemo: 100%, GEM + S-1 Radio: 0%	12
Cho et al. [26], 2013, Korea	Retro, 2002–2011	BRPC	Tumor encasement of a short segment of CHA, without evidence of tumor extension to CA; tumor abutment of the SMA involving < 180° of the circumference; or short-segment occlusion of SMV/PV, allowing for vascular reconstruction.	Chemo: 100%, GEM alone (most) or GEM + cisplatin or GEM + capecitabine Radio: 100%, 45 or 50.4 or 58.4 Gy	14
Jiang et al. [27], 2013, China	Retro, 2004–2010	RPC	Tumors not involving major vascular structures including CA, SMA, and SMV/PV.	Chemo: 72%, GEM^a^ Radio: 28%, 54 Gy	14
Patel et al. [28], 2014, America	Retro, 1995–2010	RPC/BRPC	Tumor abutment involving SMV/PV with or without narrowing or short-segment occlusion of the lumen allowing for safe resection, or tumor abutment of the SMA ≤ 180° of the circumference, or gastroduodenal artery encasement up to the hepatic artery with either short segment encasement or direct abutment of the hepatic artery, without extension to CA.	Chemo: 100%, GEM + taxotere + capecitabine Radio: 98%, 37.5 (30–50.5) Gy	12
Roland et al. [30], 2015, America	Pro, 1990–2008	RPC	No statement, but patients with borderline-resectable or locally advanced disease were excluded.	Chemo: 100%, GEM, 5- FU or capecitabine Radio: 98%, 30 or 50.4 Gy	12
Lee et al. [29], 2015, Korea	Retro, 2000–2013	RPC/BRPC	Tumor abutment (≤ 50% of the circumference) or encasement (> 50% of the circumference) of the SMV or PV.	Chemo: 100%, GEM alone (most), GEM + cisplatin or GEM + capecitabine Radio: 100%, 45 or 50.4 or 58.4 Gy	12
Sho et al. [31], 2015, Japan	Retro, 2006–2013	RPC	RPC—no tumor contact to CA, SMA, CHA, SMV/PV, or venous abutment of SMV/PV without distortion or narrowing.	Chemo: 100%, GEM Radio: 100%, 50 or 54 Gy	12
		BRPC	BRPC—tumor with encasement of a short segment of CHA without evidence of tumor extension to CA, or tumor abutment of the SMA within 180° of circumference.		
Golcher et al. [36], 2015, Germany	Pro, RCT, 2003–2009	RPC	No organ infiltration except the duodenum and maximal involvement of peripancreatic vessels ≤ 180°.	Chemo: 88%, GEM + cisplatin Radio: 88%, 50.4 Gy	Low risk of bias^b^
Hirono et al. [32], 2016, Japan	Retro, 2000–2013	BRPC	Tumor abutment of SMA within 180° of the circumference, or CHA without extension of hepatic artery bifurcation, or CA without involvement of the aorta.	Chemo: 100%, GEM + S-1 or S-1 Radio: 57%, 50 Gy	13
Masui et al. [33], 2016, Japan	Pro, 2006–2010	RPC/BRPC	Severe unilateral SMV/PV impingement, circumferential SMA abutment of less than 180°, or encasement of a short segment of the CHA.	Chemo: 100%, GEM + S-1 Radio: 0%, NA	14
Ielpo et al. [3], 2017, Spain	Pro, 2007–2016	RPC BRPC	RPC—no radiographic evidence of vascular invasion. BRPC—venous involvement of the SMV/PV; tumor abutment of the SMA within 180° of the circumference.	Chemo: 100%, GEM + nab-paclitaxel Radio: 44%, ≤ 52 Gy	15
Murakami et al. [35], 2017, Japan	Retro, 2002–2015	BRPC	Tumor contact with SMA of ≤ 180° or tumor contact with CHA without extension to the CA or hepatic artery bifurcation, allowing for safe and complete resection and reconstruction.	Chemo: 100%, GEM + S-1 Radio: 0%	13
Fujii et al. [34], 2017, Japan	Pro, 2001–2013	RPC	RPC—lesions without adjacent major vasculature including SMV/PV, SMA, CHA, and CA.	Chemo: 100%, S-1 Radio: 100%, 50.4 Gy	15
		RPC/BRPC BRPC	BR-PV—lesions involved exclusively with the SMV/PV system. BR-A—lesions involving gastroduodenal artery encasement up to the hepatic artery without extension to CA or ≤ 180° of tumor abutment to SMA.		
Jang et al. [9], 2018, Korea	Pro, RCT 2012–2014	BRPC	Tumor abutment of SMA within 180 degrees of the circumference; tumor abutment of SMV/PV with impingement and narrowing of the lumen, or short-segment venous occlusion, allowing for safe resection and reconstruction.	Chemo: 100%, GEM Radio: 100%, 45 Gy	Low risk of bias^b^
Reni et al. [12], 2018, Italy	Pro, RCT 2010–2015	RPC	Lesions with the absence of invasion of superior mesenteric artery or vein, portal vein, coeliac artery, or hepatic artery.	Chemo: 100%, cisplatin + epirubicin + capecitabine + GEM Radio: 0%	Low risk of bias^b^

*Abbreviations*: *RPC* resectable pancreatic cancer, *BRPC* borderline resectable pancreatic cancer, *Retro* retrospective, *Pro* prospective, *RCT* randomized controlled trial, *Chemo* chemotherapy, *Radio* radiotherapy, *GEM* gemcitabine, *SMV* superior mesenteric vein, *PV* portal vein, *CA* celiac axis, *CHA* common hepatic artery

^a^72% of patients only received neoadjuvant chemotherapy while 28% of patients received neoadjuvant radiotherapy alone

^b^Trials are RCTs evaluated by Cochrane Collaboration’s tool and the detailed result of assessment is showed in the Additional file 1: Table S6

**Table 2:** Summary of Clinicopathological characteristics of the eligible studies

Study	Patients factor		Tumor factor				AT, %	Matched factor^a^
	No. of participants (female, %)	Age, mean (SD), y	Size, mean (SD), cm	Site (head, %)	VR, %	CA19-9, mean (SD), U/ml		
Barbier et al. [23]	NAT: 88 SF:85	65 (39–81)^g^ 64 (37–79)	NA	100 100	16 30	> 350 (16)^b^ > 350 (15)	0 NA	1, 4, 5, 6
Papalezova et al. [24]	NAT: 144 (46) SF: 92 (47)	64 (12) 65 (12)	2.5 (1.2) 2.1 (1.3)	100 100	18 22	NA	33 66	1, 2, 4, 5
Tajima et al. [25]	NAT: 13 (46) SF: 21 (33)	63 (51–77)^g^ 66 (52–80)	NA	69 52	100 100	NA	NA	1, 2, 4, 5
Cho et al. [26]	NAT: 30 (47) SF: 21 (52)	59.57 (8.6) 60.76 (10.8)	2.6 (0.9) 2.6 (0.8)	87 86	43 38	1189 (2482) 540 (840)	50 62	1, 2, 3, 4, 5, 6, 7
Jiang et al. [27]	NAT: 112 (33) SF: 120 (43)	45.9 (9.8) 45.5 (9.3)	NA	88 80	NA	211 (46) 284 (56)	0 0	1, 2, 4, 6, 7
Patel et al. [28]	NAT: 17 (47) SF: 13 (31)	60 (39–72)^g^ 71 (42–82)	NA	88 85	NA	NA	82 77	1, 2, 4, 7
Roland et al. [30]	NAT: 222 (44) SF: 85 (40)	64 (35–86)^g^ 64 (40–85)	NA	92 87	31 27	< 1000 (74)^b^ < 1000 (66)	11 68	1, 2, 4, 5, 6
Lee et al. [29]	NAT: 30 (60) SF: 28 (50)	61.7 (8.8) 62.9 (9.6)	2.7(0.7) 2.6(0.7)	90 96	70 29	816 (1452) 504 (830)	73 75	1, 2, 3, 6, 7
Sho et al. [31]^c^	NAT: 85 (45) SF: 99 (47)	65.7 (8.9) 68.9 (10)	NA	NA	NA	NA	61 48	2, 7
Golcher et al. [36]	NAT: 33 (45) SF: 33 (48)	62.5 (33–76) 65.1 (46–73)	NA	100 100	NA 22	NA	37 30	1, 2, 4, 7
Hirono et al. [32]	NAT: 46 SF: 124	69 (41–90)^g^	3 (1.1–7.1)^g^ 2.9 (1.2–8.5)	43 56	60 42	NA	53 64	3, 4, 5, 7
Masui et al. [33]	NAT: 18 (56) SF: 19 (68)	63 (43–73)^g^ 66 (56–80)	3.3 (1.8–5)^g^ 3.2 (1.7–7.5)	72 68	47 37	102 217	78 84	1, 2, 3, 4, 5, 6, 7
Ielpo et al. [3]^d^	NAT: 45 (36) SF: 36 (42)	62 (42–81)^f^ 64 (46–78)	7.5 6.8	71 58	35 36	1754 1621	61 58	1, 2, 3, 4, 5, 6, 7
Murakami et al	NAT: 52 (33) SF: 25 (28)	> 67 (48)^b^ > 67 (60)	≥ 37 (52)^b^ ≥ 37 (34)	67 84	62 57	> 150 (52)^b^ > 150 (52)	80 48	1, 2, 4, 6
Fujii et al. [34]^e^	NAT: 40 (48) SF: 233 (37)	65 (36–79)^g^ 67 (35–88)	2.9 (1.5–5.2)^g^ 2.5 (0.8–5.6)	100 100	25 32	143 148	67 66	1,2,3,4,5,6,7
	NAT: 27 (56) SF: 102 (48)	68 (47–78)^g^ 66 (39–83)	3 (1.8–3.9)^g^ 3.3 (1.5–7)	100 100	96 95	323 259	39 44	1, 2, 3, 4, 5, 6, 7
	NAT: 21 (52) SF: 81 (37)	68 (47–76)^g^ 65 (42–82)	3.5 (2.6–4.6)^g^ 3 (2–6)	100 100	79 87	286 218	46 43	1, 2, 3, 4, 5, 6, 7
Jang et al. [9]	NAT: 27 (37) SF: 23 (35)	59.4 (8.4) 58.9 (11.3)	3.4 (0.8) 3.5 (0.9)	85 74	35 28	1042 (2465) 1258 (2540)	52 57	1, 2, 3, 4, 5, 6, 7
Reni et al. [12]	NAT: 32 (22) SF: 26 (46)	64 (39–75)^g^ 65 (37–74)	2.0 (0–6.0)^g^ 2.5 (1.5–5.0)	88 96	0 9	173 (43–4510) 179 (39–3337)	72 65	1, 2, 3, 4, 5, 6, 7

*Abbreviations*: *NAT* neoadjuvant therapy, *SF* surgery first, *AT* adjuvant therapy, *VR* vascular resection, *NA* not available

^a^Factors matched with NAT and SF: 1, age; 2, sex; 3, initial tumor size; 4, tumor location; 5, vascular resection; 6, initial CA19-9 level; 7, AT

^b^Reported as range (percentage, %)

^c^The study by Sho et al. has 2 independent data sets (1 data set for RPC and 1 data set for BRPC)

^d^The study by lelpo et al. has 1 data set for RPC/BRPC, but it has 2 data subsets (1 data subset for RPC and 1 data subset for BRPC)

^e^The study by Fujii et al. has 3 independent data sets (1 data set for RPC, 1 data set for BRPC, and 1 data set for RPC/BRPC)

^f^Reported as mean (range)

^g^Reported as median (range)

### Overall survival

**Fig. 2:**
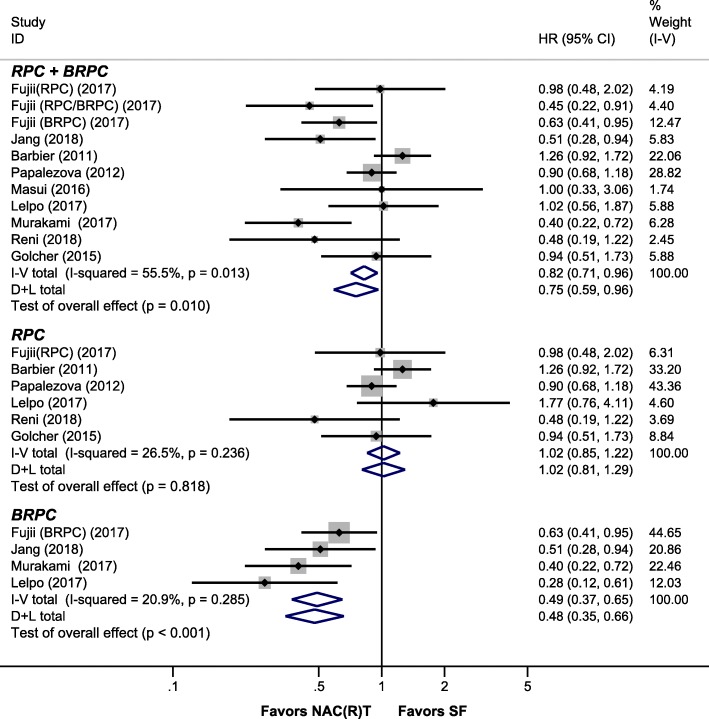
Pooled HR for OS in intention-to-treat analysis. Abbreviations: RPC, resectable pancreatic cancer; BRPC, borderline resectable pancreatic cancer; NAC(R)T, neoadjuvant chemo(radio)therapy; SF, surgery first

Firstly, we did an intention-to-treat (ITT) pooled analysis, which means both NAC(R)T and SF groups included patients who did not undergo surgery. Nine studies (11 data sets) [3, 9, 12, 23, 24, 33–36] presented the data in ITT analysis, and the pooled analysis suggested NAC(R)T had significantly better OS than SF (HR, 0.75 [95% CI, 0.59–0.96], *I*^2^ = 55.5%) for RPC + BRPC patients. According to the resectability status, RPC patients had similar OS between NAC(R)T and SF (HR, 1.02 [95% CI, 0.85–1.22], *I*^2^ = 26.5%). For BRPC patients, significantly better OS was shown after NAC(R)T (HR, 0.48 [95% CI, 0.35–0.66], *I*^2^ = 20.9%) (Fig. 2).

**Fig. 3:**
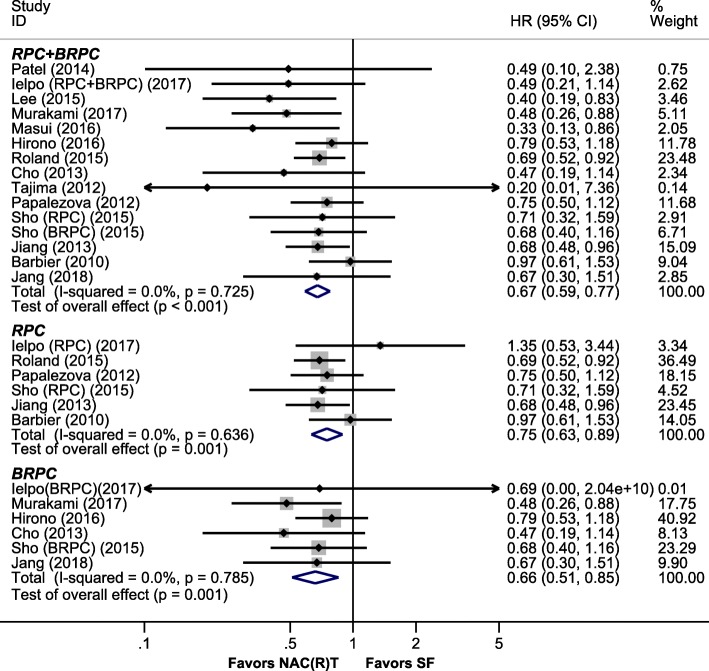
Pooled HR for OS in resected patients. Abbreviations: RPC, resectable pancreatic cancer; BRPC, borderline resectable pancreatic cancer; NAC(R)T, neoadjuvant chemo(radio)therapy; SF, surgery first

Secondly, 14 studies (15 data sets) [3, 9, 23–33, 35] presented the data of resected patients, and the results demonstrated that NAC(R)T had significantly better OS compared to SF (HR, 0.67 [95% CI, 0.59–0.77], *I*^2^ = 0%) for resected RPC + BRPC patients. Based on resectability status, NAC(R)T showed significantly better OS than SF for resected patients with RPC (HR, 0.75 [95% CI, 0.63–0.89], *I*^2^ = 0%) or BRPC (HR, 0.66 [95% CI, 0.51–0.85], *I*^2^ = 0%) (Fig. 3).

### 1-, 3-, and 5-year survival rates

**Fig. 4:**
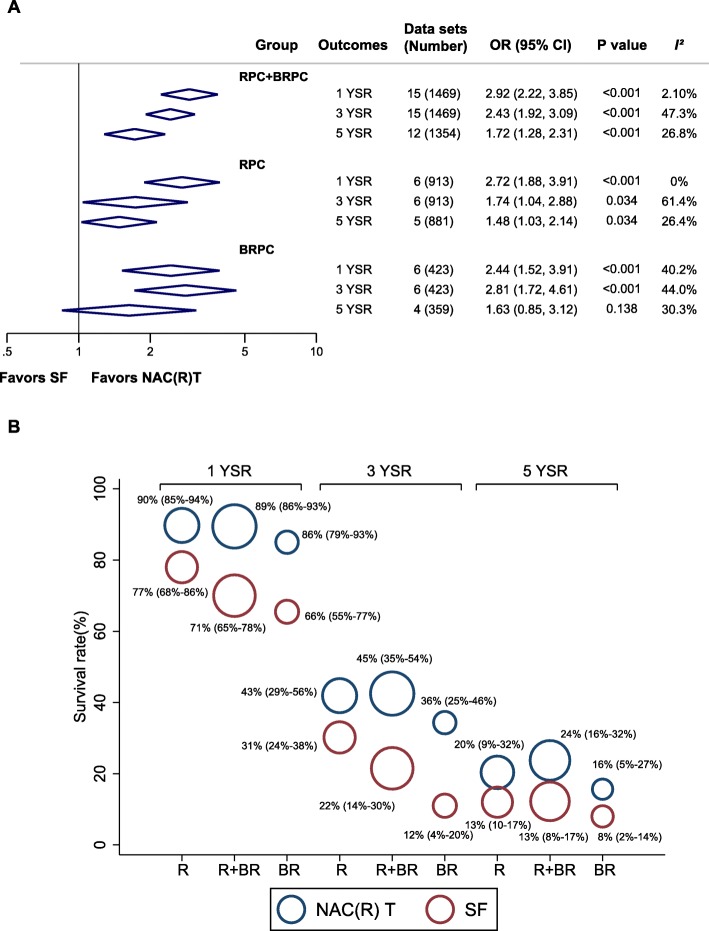
Summary of 1-, 3-, and 5-year survival rates in resected patients. **a** Forest plot of meta-analysis. **b** Bubble plot using individual hospital data sets. Sizes of circles are proportional to the number of cases. Numbers in parenthesis indicate 95% CIs. Abbreviations: R or RPC, resectable pancreatic cancer; BR or BRPC, borderline resectable pancreatic cancer; NAC(R)T, neoadjuvant chemo(radio)therapy; SF, surgery first; NA, not applicable; YSR, year survival rate

Figure 4a displays the pooled results of 1-, 3-, and 5-YSRs in resected patients. The pooled outcomes indicated that resected RPC + BRPC patients that underwent NAC(R)T had higher 1-, 3-, and 5-YSRs than SF (OR, 2.92 [95% CI, 2.22–3.85], *I*^2^ = 2.1%; OR, 2.43 [95% CI, 1.92–3.09], *I*^2^ = 47.3%; OR, 1.72 [95% CI, 1.28–2.31], *I*^2^ = 26.8%, respectively). Based on resectability status, NAC(R)T showed significantly higher 1-, 3-, and 5-YSRs than SF for resected patients with RPC or BRPC (all *P* ≤ 0.034, *I*^2^ range from 0 to 61.4%), except 5-YSR in BRPC patients (OR, 1.63 [95% CI, 0.85–3.12], *I*^2^ = 30.3%).

Figure 4b showed the mean 1-, 3-, and 5-YSRs following NAC(R)T and SF, in which the size of circles represents the number of cases in each study. For resected RPC + BRPC, the mean 1-, 3-, and 5-YSRs after NAC(R)T were 89%, 45%, and 24% and those after SF were 71%, 22%, and 13%, respectively. As for resectability status, similar trends were observed in RPC and BRPC.

### Sensitivity analysis, subgroup analysis, and meta-regression analysis

All the sensitivity analyses for OS and representative 3-YSR in resected patients are summarized in Additional file 1: Table S7. Sensitivity analyses including matched patient factors, matched tumor size, matched vascular resection, matched CA19-9 level, matched tumor factors, matched patient and tumor factors, matched pancreatic head cancer (≥ 80% of patients), matched AT, and Asians demonstrated that an improvement in mortality after NAC(R)T over SF were consistent with the evidence from primary outcomes analysis, except in RPC or BRPC with matched tumor sizes, matched tumor factors, and matched patient and tumor factors (*P* > 0.05). The eligible data sets with matched relevant factors above were insufficient (≤ 3), inevitably reducing the reliability of results.

**Fig. 5:**
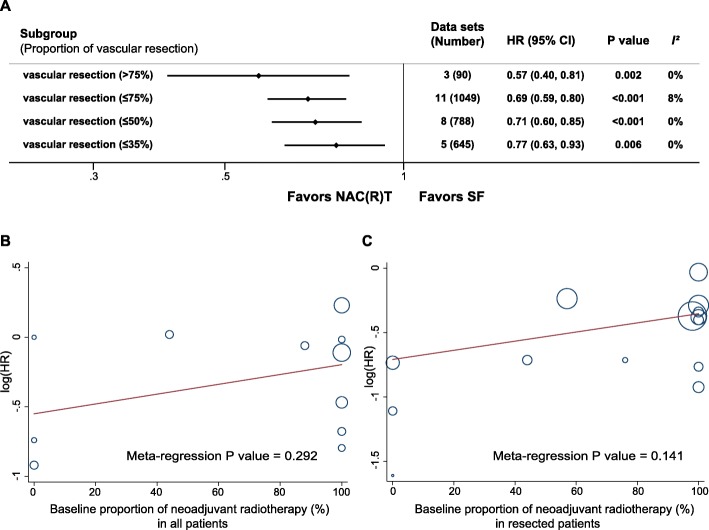
Results of subgroup and meta-regression analyses. **a** Subgroup analysis. **b** Meta-regression analysis in all patients. **c** Meta-regression analysis in resected patients. Abbreviations: NAC(R)T, neoadjuvant chemo(radio)therapy; SF, surgery first

The subgroup analysis according to proportion of concomitant vascular resection is shown in Fig. 5a. For RPC + BRPC, the survival benefits for NAC(R)T over SF are consistent across different vascular resection proportion (all *P* < 0.01). Moreover, the pooled results for the studies including > 75% proportion of vascular resection (HR, 0.57 [95% CI, 0.40–0.81], *I*^2^ = 0%) tended to more favor NAC(R)T than those results for < 75% proportion of vascular resection (HR, 0.69 [95% CI, 0.59–0.80], *I*^2^ = 8.0%), < 50% proportion of vascular resection (HR, 0.71 [95% CI, 0.46–0.65], *I*^2^ = 0%), and < 35% proportion of vascular resection (HR, 0.77 [95% CI, 0.63–0.93], *I*^2^ = 0%).

Meta-regression analysis indicated that additional neoadjuvant radiotherapy had little effects on the pooled HR for OS comparing NAC(R)T with SF in all the patients and resected patients (all *P* > 0.05, Fig. 5b, c).

### Second outcomes and publication bias

All of second outcomes are shown in Table 3. NAC(R)T had significantly better DFS compared to SF for RPC + BRPC (HR, 0.66 [95% CI, 0.53–0.83], *I*^2^ = 0%, *P* < 0.001). According to resectability status, BRPC showed significantly better DFS after NAC(R)T than SF (HR, 0.44 [95% CI, 0.26–0.73], *I*^2^ = 0%). There is no statistical difference for DFS in RPC (HR, 0.80 [95% CI, 0.59–1.07], *I*^2^ = 0%), but the tendency was not changed. The recurrence rate was lower in BRPC that underwent NAC(R)T (OR, 0.41 [95% CI, 0.22–0.76], *I*^2^ = 10.2%) while it is similar between the two methods in RPC (OR, 0.77 [95% CI, 0.55–1.08], *I*^2^ = 0%). The overall resection rate was not statistically different between the two treatment modalities in BRPC (OR, 0.69 [95%, 0.41–1.16], *I*^2^ = 36.1%), but RPC that underwent NAC(R)T had lower resection rate than SF (OR, 0.50 [95%, 0.25–0.99], *I*^2^ = 60.4%). R0 rates and pN+ rates are in support of NAC(R)T regardless of resectability status (all *P* < 0.05).

**Table 3:** Summary of second outcomes in this meta-analysis

Outcome of interest	RPC + BRPC				RPC				BRPC			
	Data sets	ES (95% CIs)	*P* value	*I*^2^ (%)	Data sets	ES (95% CIs)	*P* value	*I*^2^ (%)	Data sets	ES (95% CIs)	*P* value	*I*^2^ (%)
DFS	7	0.66 (0.53–0.83)	< 0.001	0	3	0.80 (0.59–1.07)	0.137	0	2	0.44 (0.26–0.73)	0.002	0
Overall resection rate	12	0.64 (0.37–1.10)	0.104	60.0	6	0.50 (0.25–0.99)	0.048	60.4	5	0.69 (0.41–1.16)	0.159	36.1
R0 rate	19	2.83 (2.19–3.65)	< 0.001	40.2	8	1.95 (1.40–2.71)	< 0.001	22.3	6	4.75 (2.85–7.92)	< 0.001	16.4
Recurrence	12	0.65 (0.50–0.86)	0.003	0	4	0.77 (0.55–1.08)	0.131	0	5	0.41 (0.22–0.76)	0.005	10.2
pN+ rate	18	0.30 (0.20–0.43)	< 0.001	58.4	7	0.28 (0.21–0.38)	< 0.001	0	6	0.23 (0.07–0.75)	0.015	82.8

*DFS* disease-free survival, *pN+* pathological positive lymph node, *ES* indicates effect size

The funnel plots of OS comparing NAC(R)T with SF in all patients and resected patients were shown in Additional file 1: Figure S1. No significant asymmetry of the funnel plots was detected, except the funnel plot for resected RPC + BRPC (Begg’s *P* = 0.023, Egger’s *P* = 0.018) (Additional file 1: Table S8). Therefore, we conducted a sensitivity analysis using the trim and fill method [39]. Interestingly, a symmetrical funnel plot was produced without hypothetical studies filled. It means that the new funnel plot was just the original graph and the pooled results was reliable although there was a possibility of publication bias in pooled OS in resected RPC + BRPC.

## Discussion

This meta-analysis with 2286 (1082 vs 1204) patients only included comparative trials from 2011 to 2018 and mainly focus on survival outcomes between NAC(R)T and SF for resectable or borderline resectable PDAC. In ITT analysis, BRPC patients who underwent NAC(R)T have increased OS in comparison to SF while similar OS was observed between NAC(R)T and SF in RPC patients. In resected patients, NAC(R)T markedly increase OS, and 1-, 3-, and 5-YSRs compared to SF regardless of patients with RPC or BRPC.

Recently, there is one single-arm meta-analysis published in 2018 by Versteijne et al. [11] containing several single-arm trials besides comparative trials, which found that neoadjuvant treatment improved median OS by ITT analysis in resectable or borderline resectable PDAC (RPC + BRPC, 18.8 vs 14.8 months; BRPC, 19.2 vs 12.8 months). However, comparing with their study, our study only included comparative trials using HR to analyze the survival benefits between SF and NAC(R)T and found that NAC(R)T has no significant advantages in resectable PDAC in comparison to SF by ITT analysis (HR = 1.02, *P* = 0.818), which was consistent with their results (median OS in RPC, 18.2 vs 17.7 months).

For BRPC patients, a higher OS was shown in NAC(R)T group regardless of the analysis of all patients (HR = 0.49, *P* < 0.001) or resected patients (HR = 0.66, *P* = 0.001). Besides, patients who underwent NAC(R)T had higher DFS, lower recurrence rate, higher R0 rate, and similar overall resection rate compared with patients who underwent SF (DFS: HR = 0.44, *P* = 0.002; recurrence rate: OR = 0.41, *P* = 0.005; R0 rate: OR = 4.75, *P* < 0.001; overall resection rate: NAT, 76%; SF, 81%; OR = 0.69, *P* = 0.159). Based on the data above, NAC(R)T can provide survival benefits in BRPC patients in comparison to SF, which should be considered as the preferred method for the management of BRPC in the real world.

For RPC patients, NAC(R)T has a similar OS in ITT analysis but a higher OS in the analysis of resected patients compared with SF (HR = 0.75, *P* = 0.001). Moreover, RPC patients who underwent NAC(R)T had higher DFS and lower recurrence rate than those who underwent SF, although advantages did not reach statistical significance (DFS: HR = 0.80, *P* = 0.137; recurrence rate: OR = 0.77, *P* = 0.131). Also, R0 rate in NAC(R)T is higher than SF (NAT, 89%; SF, 78%; OR 1.95, *P* < 0.001), but overall resection rate in NAC(R)T is lower than SF (NAT, 66%; SF, 81%; OR 0.50, *P* = 0.048). Our study indicated that there may exist a subgroup of RPC patients who are sensitive to chemo(radio)therapy and can obtain survival benefits from neoadjuvant therapy. Therefore, looking for potential biomarkers to screen patients who can benefit from NAC(R)T is urgent in the future.

Furthermore, it is a pity that the eligible data sets are so insufficient that we are unable to compare OS of patients who received NAC(R)T followed by resection with those who received SF followed by AT (SF + AT). Mokdad et al. [10] using a national cohort from National Cancer Database (2006–2012) found that the survival benefits were maintained in the NAC(R)T group in comparison with SF + AT for resected RPC patients (HR, 0.83 [95% CI, 0.78–0.89]). Similar result was also found by Parmar et al. [38] using the data from Surveillance, Epidemiology, and End Results database for resected RPC patients without vascular invasion (HR, 0.54 [95% CI, 0.40–0.72]). However, the only one RCT performed by Jang et al. [9] has reported that there was no significant difference in OS between NAC(R)T group and SF + AT group for resected BRPC patients (HR 0.67 [95% CI, 0.30–1.52]) with a total of 29 patients (17 vs 12). Given that the small sample size in the study by Jang et al. [9], we consider the trend is the same but the 95% CI is wide. Moreover, although the additional chemotherapy after surgery has been shown to improve OS, the implementation of AT is limited by performance status of patients, postoperative complications, and early disease progression [40–42]. Of course, AT is still recommended after NAC(R)T followed by resection as long as patients can tolerate postoperative chemotherapy [40].

For patients with RPC or BRPC, vascular resection with concomitant reconstruction is widely used to attain negative margins during the pancreatic resection. Currently, pancreaticoduodenectomy combined with venous resection is proved to be safe and feasible and has the same long-term survival if R0 resection can be achieved [43–45]. Our subgroup analysis further found RPC + BRPC patients with a higher baseline proportion of vascular resection tended to show more survival benefits for NAC(R)T over SF (> 75% of vascular resection vs ≤ 35% of vascular resection; HR, 0.57 vs 0.77, respectively). Lee et al. [29] also found NAC(R)T achieved better survival outcomes than SF in RPC + BRPC with vascular resection. Therefore, NAC(R)T should be considered as a preferred therapeutic strategy for patients who may require vascular resection in the preoperative evaluation, especially in BRPC patients.

There are various chemoradiotherapy regimens in this meta-analysis, including multiple-agents chemotherapy (4 trials), combined single-agent chemotherapy and radiotherapy (8 trials), and combined multiple-agents chemotherapy and radiotherapy (5 trials), which is inherent heterogeneity in our study. Accordingly, the outcomes should be explained cautiously. At present, a number of RCTs are ongoing comparing survival benefits between neoadjuvant therapy based on more effective regimens and immediate surgery, which will provide more evidence about the role of neoadjuvant therapy in the treatment of RPC (NCT02172976, NCT02047513, and NCT02919787).

As for variation in the dose of radiotherapy, meta-regression analyses were used to assess the effect of additional preoperative radiotherapy on the survival benefits and the result showed, relative to neoadjuvant chemotherapy alone, no significant differences in OS were found both in all patients and resected patients (all *P* > 0.05). Besides, the study by Cloyd et al. [46] also indicated that a high-dose (50.4 Gy) radiotherapy combined with chemotherapy was associated with similar OS in comparison with a standard dose (30 Gy) chemoradiotherapy or chemotherapy alone in patients undergoing pancreatectomy for PDAC in multivariate cox regression analysis. Meanwhile, several RCTs are in progress to investigate the survival benefits of different neoadjuvant regimens for the treatment of BRPC or RPC, contributing to determining the optimal chemoradiotherapy regimens (NCT02562716 and NCT03777462).

This study has several limitations. First, the majority of evidence in favor of NAC(R)T are based on NRCTs that increase the risk of potential selection and publication bias. However, considering that NRCTs usually have large sample sizes, a meta-analysis of RCTs is not necessarily superior to well-designed NRCTs in terms of evidence level [47]. In our study, all the included literatures were relatively high quality (modified MINORS score ≥ 12) indicating a low risk of bias. Besides, the between-study heterogeneity on most outcomes was low. Also, elaborate sensitivity, subgroup, and meta-regression analyses had demonstrated the stability of outcomes. Second, heterogeneity exists in chemotherapy regimen and radiotherapy dose, as discussed previously, so results should be interpreted with caution. Therefore, more large-scale and well-designed RCTs with more effective regimens are needed to investigate survival outcome between NAC(R)T and SF in resectable PDAC.

## Conclusions

This meta-analysis uses stratified analysis as well as sophisticated subgroup and sensitivity analyses to demonstrate that NAC(R)T can provide survival benefits in patients with BRPC and a subgroup of RPC in comparison with SF. Future researches should look for potential biomarkers to screen the subgroup of RPC patients who can benefit from neoadjuvant therapy.

## Supplementary information

**Additional file 1: Table S1.** Search Strategy for Each Database. **Table S2.** Inclusion and Exclusion criteria. **Table S3.** Modified Methodological Index for Non-Randomized Studies (MINORS) score for nonrandomized comparative studies. **Table S4.** Criteria defining resectability status in NCCN guideline version 2.2018. **Table S5.** Modified Methodological Index for Non-Randomized Studies (MINORS) score for assessing the quality of all eligible nonrandomized comparative studies. **Table S6.** Risk of Bias Assessment using Cochrane Collaboration’s tool in the randomized controlled trial included in the Meta-Analysis. **Table S7.** Summary of Sensitivity Analysis for Overall Survival and 3-Year Survival Rate in Resected patients. **Table S8.** Quantitative Assessment for Asymmetry of Funnel Plots. **Figure S1.** Funnel Plots of Overall Survival in all the patients (A-C) and resected patients (D-G).

## Data Availability

All data generated or analyzed during this study are included in this published article.

## Abbreviations

BRPC: Borderline resectable pancreatic cancer
CI: Confidence intervals
DFS: Disease-free survival
HR: Hazard ratio
NAC(R)T: Neoadjuvant chemo(radio)therapy
NRCTs: Non-randomized comparative trials
ORs: Odds ratios
OS: Overall survival
PRISMA: Preferred Reporting Items for Systematic Reviews and Meta-Analyses
R0 rate: Completeness of resection
RPC: Resectable pancreatic cancer
SF: Surgery first
YSR: Year survival rate
