# Supplementary material for: Survival benefits of neoadjuvant chemo(radio)therapy versus surgery first in patients with resectable or borderline resectable pancreatic cancer: a systematic review and meta-analysis
Source: World J Surg Oncol. 2019 Dec 31;18:1. doi: 10.1186/s12957-019-1767-5 (PMC6937851; doi:10.1186/s12957-019-1767-5)
Supplement: Supplementary file 1 — Additional file 1: Table S1. Search Strategy for Each Database. Table S2. Inclusion and Exclusion criteria. Table S3. Modified Methodological Index for Non-Randomized Studies (MINORS) score for nonrandomized comparative studies. Table S4. Criteria defining resectability status in NCCN guideline version 2.2018. Table S5. Modified Methodological Index for Non-Randomized Studies (MINORS) score for assessing the quality of all eligible nonrandomized comparative studies. Table S6. Risk of Bias Assessment using Cochrane Collaboration’s tool in the randomized controlled trial included in the Meta-Analysis. Table S7. Summary of Sensitivity Analysis for Overall Survival and 3-Year Survival Rate in Resected patients. Table S8. Quantitative Assessment for Asymmetry of Funnel Plots. Figure S1. Funnel Plots of Overall Survival in all the patients (A-C) and resected patients (D-G). [file 12957_2019_1767_MOESM1_ESM.docx]

**Supplementary material**

**Contents**

**Table S1.** Search Strategy for Each Database.

**Table S2.** Inclusion and Exclusion criteria.

**Table S3.** Modified Methodological Index for Non-Randomized Studies (MINORS) score for nonrandomized comparative studies.

**Table S4.** Criteria defining resectability status in NCCN guideline version 2.2018.

**Table** **S5.** Modified Methodological Index for Non-Randomized Studies (MINORS) score for assessing the quality of all eligible nonrandomized comparative studies.

**Table S6.** Risk of Bias Assessment using Cochrane Collaboration’s tool in the randomized controlled trial included in the Meta-Analysis.

**Table S7.** Summary of Sensitivity Analysis for Overall Survival and 3-Year Survival Rate in Resected patients.

**Table S8.** Quantitative Assessment for Asymmetry of Funnel Plots.

**Figure S1.** Funnel Plots of Overall Survival in all the patients (A-C) and resected patients (D-G).

**Table S1. Search Strategy for Each Database.**

| **Database** | **Search strategy** |
| --- | --- |
| PubMed | #1 (surgery[Title/Abstract] OR resection[Title/Abstract]) |
|  | #2 "Neoadjuvant Therapy"[Mesh] |
|  | #3 neoadjuvant[Title/Abstract] |
|  | #4 #2 AND #3 |
|  | #5 pancreatic[Title/Abstract] |
|  | #6 cancer[Title/Abstract] OR adenocarcinoma[Title/Abstract]) OR carcinoma[Title/Abstract]) OR neoplasm[Title/Abstract] |
|  | #7 #5 AND #6 |
|  | #8 "Pancreatic Neoplasms"[Majr] |
|  | #9 #7 OR #8 |
|  | #10 #1 AND #4 AND #9 |
|  | #11 review[Publication Type] |
|  | #12 systematic[Title/Abstract] OR meta-analysis[Title/Abstract]) OR pooled[Title/Abstract]) OR pooling[Title/Abstract] |
|  | #13 #11 NOT #12 |
|  | #14 #10 NOT #13 |
|  | #15 "humans"[MeSH Terms] |
|  | #16 #14 AND #15 |
| EMBASE | #1 'neoplasm':ti,ab,kw OR 'carcinoma':ti,ab,kw OR 'adenocarcinoma':ti,ab,kw OR 'cancer':ti,ab,kw |
|  | #2 'pancreatic':ti,ab,kw |
|  | #3 #1 AND #2 |
|  | #4 'pancreas adenocarcinoma'/mj |
|  | #5 #3 OR #4 |
|  | #6 'neoadjuvant':ti,ab,kw |
|  | #7 'neoadjuvant therapy'/exp |
|  | #8 #6 OR #7 |
|  | #9 'surgery':ti,ab,kw OR 'resection':ti,ab,kw |
|  | #10 #5 AND #8 AND #9 AND ([article]/lim OR [article in press]/lim) AND [english]/lim AND [humans]/lim AND [embase]/lim |
| Web of Science | #1 TOPIC: (neoplasm) OR TOPIC: (carcinoma) OR TOPIC: (adenocarcinoma) OR TOPIC: (cancer) |
|  | #2 TOPIC: (pancreatic) |
|  | #3 #2 AND #1 |
|  | #4 TOPIC: (neoadjuvant) |
|  | #5 TOPIC: ("upfront resection") OR TOPIC: ("primary surgery") OR TOPIC: ("primary resection") OR TOPIC: ("immediate surgery") OR TOPIC: ("surgery first") |
|  | #6 #5 AND #4 AND #3 |

**Table S1. Search Strategy for Each Database (continued).**

| **Database** | **Search strategy** |
| --- | --- |
| Cochrane library | #1 "surgery":ti,ab,kw or "resection":ti,ab,kw (Word variations have been searched) |
|  | #2 Neoadjuvant:ti,ab,kw (Word variations have been searched) |
|  | #3 MeSH descriptor: [Neoadjuvant Therapy] explode all trees |
|  | #4 #2 or #3 |
|  | #5 neoplasm:ti,ab,kw or carcinoma:ti,ab,kw or adenocarcinoma:ti,ab,kw or cancer:ti,ab,kw (Word variations have been searched) |
|  | #6 "pancreatic":ti,ab,kw (Word variations have been searched) |
|  | #7 #5 and #6 |
|  | #8 MeSH descriptor: [Pancreatic Neoplasms] explode all trees |
|  | #9 #7 or #8 |
|  | #10 #1 and #4 and #9 |

**Table S2. Inclusion and Exclusion criteria.**

| **Inclusion criteria** | (1) studies involving patients with resectable or borderline resectable pancreatic adenocarcinoma (based on histological results). |
| --- | --- |
|  | (2) studies comparing neoadjuvant chemotherapy with or without radiotherapy to surgery first with or without adjuvant therapy. |
|  | (3) studies providing the results of overall survival. |
|  | (4) studies design including randomized controlled trials and non-randomized comparative trials. |
| **Exclusion criteria** | (1) studies containing other cancer cases like cystadenocarcinoma, neuroendocrine neoplasia and ampullary cancer |
|  | (2) Different resectability status of patients between experiment group and control group |
|  | (3) studies with no detailed or estimable resectability classification |
|  | (4) Non-comparative studies including abstracts, conference articles, opinions, case reports, and reviews. |

**Table S3. Modified Methodological Index for Non-Randomized Studies (MINORS) score for non-randomized comparative studies.**

| **Items** | **Points** | | |
| --- | --- | --- | --- |
|  | **0** | **1** | **2** |
| Consecutive patients | Not reported | Patients not consecutive | Patients consecutive |
| Prospective data collection | Not reported | Data obtained from retrospective review of medical history | Data obtained from prospectively maintained database |
| Reported primary endpoints | Not reported | Only secondary or incomplete primary outcome reported | Complete primary outcomes reported |
| Unbiased post-procedural evaluation | No protocol for peri-procedural clinical management or therapy used to treat recurrence | Incomplete protocol for peri-procedural clinical management or therapy used to treat recurrence | Complete protocol for peri-procedural clinical management or therapy used to treat recurrence |
| Appropriate control intervention | Not reported | Incomplete report of the standard intervention | Complete report of the standard intervention |
| Contemporary groups | Not reported | Study group compared with historical control group | Study group compared with contemporary control group |
| Group equivalent | No matching analysis performed | Matching incomplete | Matching complete for patient factors (age, sex) and tumor factors (tumor size, tumor location, vascular resection, initial CA19-9 level). |
| sample size | <20 of each group | 20–50 of each group | >50 of each group |

**Table S4.** **Criteria defining resectability status in NCCN guideline version 2.2018.**

| **Resectability Status** | **Arterial** | **Venous** |
| --- | --- | --- |
| **Resectable** | No arterial tumor contact (celiac axis [CA], superior mesenteric artery [SMA], or common hepatic artery [CHA]). | No tumor contact with the superior mesenteric vein (SMV) or portal vein (PV) or ≤180° contact without vein contour irregularity. |
| **Borderline Resectable** | **Pancreatic head/uncinate process:**  • Solid tumor contact with CHA without extension to CA or hepatic artery bifurcation allowing for safe and complete resection and reconstruction.  • Solid tumor contact with the SMA of ≤180°  • Solid tumor contact with variant arterial anatomy (ex: accessory right hepatic artery, replaced right hepatic artery, replaced CHA, and the origin of replaced or accessory artery) and the presence and degree of tumor contact should be noted if present, as it may affect surgical planning.  **Pancreatic body/tail:**  • Solid tumor contact with the CA of ≤180°  • Solid tumor contact with the CA of >180° without involvement of the aorta and with intact and uninvolved gastroduodenal artery thereby permitting a modified Appleby procedure [some panel members prefer these criteria to be in the unresectable category]. | • Solid tumor contact with the SMV or PV of >180°, contact of ≤180° with contour irregularity of the vein or thrombosis of the vein but with suitable vessel proximal and distal to the site of involvement allowing for safe and complete resection and vein reconstruction.  • Solid tumor contact with the inferior vena cava (IVC). |

**Table S5. Modified Methodological Index for Non-Randomized Studies (MINORS) score for Assessing the quality of all eligible nonrandomized comparative studies.**

| **study(year)** | **Consecutive patients** | **Prospective data collection** | **Reported primary endpoints** | **Unbiased post-procedural evaluation** | **Appropriate control intervention** | **Contemporary groups** | **Group equivalent** | **Sample size** | **Total score** |
| --- | --- | --- | --- | --- | --- | --- | --- | --- | --- |
| Ielpo et al (2017) | 2 | 2 | 2 | 2 | 2 | 2 | 2 | 1 | 15 |
| Lee et al (2015) | 1 | 1 | 2 | 2 | 2 | 2 | 1 | 1 | 12 |
| Murakami et al (2017) | 2 | 1 | 2 | 2 | 2 | 2 | 1 | 1 | 13 |
| Masui et al (2016) | 2 | 2 | 2 | 2 | 2 | 2 | 2 | 0 | 14 |
| Hirono et al (2016) | 2 | 1 | 2 | 2 | 2 | 2 | 1 | 1 | 13 |
| Roland et al (2015) | 1 | 2 | 2 | 2 | 1 | 1 | 1 | 2 | 12 |
| Cho et al (2013) | 2 | 1 | 2 | 2 | 2 | 2 | 2 | 1 | 14 |
| Tajima et al (2012) | 2 | 1 | 2 | 2 | 2 | 2 | 1 | 0 | 12 |
| Papalezova et al (2012) | 1 | 1 | 2 | 2 | 2 | 2 | 1 | 2 | 13 |
| Fujii et al (2017) | 2 | 2 | 2 | 2 | 2 | 2 | 2 | 1 | 15 |
| Sho et al (2015) | 2 | 1 | 2 | 2 | 2 | 2 | 0 | 1 | 12 |
| Patel et al (2014) | 1 | 1 | 2 | 2 | 1 | 2 | 1 | 2 | 12 |
| Jiang et al (2013) | 2 | 1 | 2 | 2 | 2 | 2 | 1 | 2 | 14 |
| Barbier et al (2011) | 2 | 1 | 2 | 2 | 2 | 2 | 1 | 2 | 14 |

**Table S6. Risk of Bias Assessment using Cochrane Collaboration’s tool in the randomized controlled trial included in the Meta-Analysis.**

**
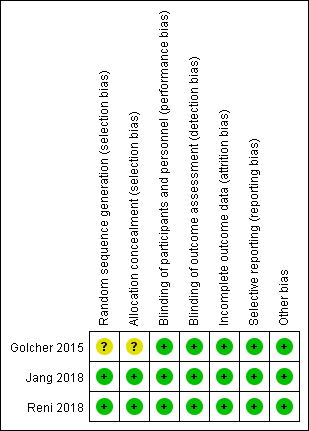
**

**Table S7. Summary of Sensitivity Analysis for Overall Survival and 3-Year Survival Rate in Resected patients**

|  |  | **Overall Survival** | | | **3-Year Survival Rate** | | |
| --- | --- | --- | --- | --- | --- | --- | --- |
| **Variables** | **Data Sets** | **HR (95% CI)** | **P Value** | ***I^2^*** | **OR (95% CI)** | **P Value** | ***I^2^*** |
| **RPC+BRPC** | | | | | | | |
| Exclusion of Jiang 2013 | 14 (1237) | 0.67 (0.58-0.78) | <0.001 | 0% | 2.21 (1.70-2.89) | <0.001 | 43.7% |
| Matched patient factors^a^ | 11 (1033) | 0.62 (0.53-0.74) | <0.001 | 0% | 2.90 (2.18-3.86) | <0.001 | 41.3% |
| Matched tumor size | 6 (373) | 0.59 (0.45-0.78) | <0.001 | 2.3% | 4.23 (2.49-7.19) | <0.001 | 4.7% |
| Matched vascular resection | 9 (891) | 0.71 (0.60-0.84) | <0.001 | 0% | 1.94 (1.42-2.66) | <0.001 | 32.8% |
| Matched initial CA19-9 level | 9 (944) | 0.65 (0.55-0.76) | <0.001 | 8.6% | 2.86 (1.55-5.27) | 0.001 | 60.9% |
| Matched tumor factors^b^ | 4 (172) | 0.49 (0.32-0.76) | 0.001 | 0% | 5.00 (2.31-10.85) | <0.001 | 28.7% |
| Matched patient and tumor factors | 4 (303) | 0.49 (0.32-0.76) | 0.001 | 0% | 3.48 (2.03-5.98) | <0.001 | 16.9% |
| Pancreatic head cancer (≥80% of patients) | 8^c^ (947) | 0.70 (0.59-0.82) | <0.001 | 0% | 2.39 (1.43-4.00) | 0.001 | 59.0% |
| Matched adjuvant therapy | 10 (823) | 0.63 (0.52-0.77) | <0.001 | 0% | 3.32 (2.41-4.58) | <0.001 | 27.5% |
| Asians | 10 (816) | 0.63 (0.52-0.76) | <0.001 | 0% | 3.03 (2.19-4.19) | <0.001 | 0% |
| **RPC** | | | | | | | |
| Except Jiang 2013 | 5 (681) | 0.77 (0.64-0.94) | 0.010 | 0% | 1.49 (1.06-2.09) | 0.023 | 27.4% |
| Matched patient factors^a^ | 4 (715) | 0.72 (0.59-0.87) | 0.001 | 0% | 2.00 (1.17-3.41) | 0.011 | 56.2% |
| Matched tumor size | 1 (32) | 1.35 (0.53-3.44) | 0.529 | NA | 0.95 (0.23-3.92) | 0.946 | NA |
| Matched vascular resection | 4 (676) | 0.78 (0.64-0.95) | 0.014 | 0% | 1.36 (0.94-1.98) | 0.102 | 1.9% |
| Matched initial CA19-9 level | 4 (676) | 0.75 (0.62-0.91) | 0.004 | 11.7% | 1.54 (0.72-3.29) | 0.270 | 74% |
| Matched tumor factors^b^ | 1 (32) | 1.35 (0.53-3.44) | 0.529 | NA | 0.95 (0.23-3.92) | 0.946 | NA |
| Matched patient and tumor factors | 1 (32) | 1.35 (0.53-3.44) | 0.529 | NA | 0.95 (0.23-3.92) | 0.946 | NA |
| Pancreatic head cancer (≥80% of patients) | 4 (788) | 0.74 (0.62-0.88) | 0.001 | 0% | 1.74 (0.93-3.26) | 0.084 | 71.8% |
| Matched adjuvant therapy | 3 (357) | 0.73 (0.54-1.00) | 0.047 | 0% | 2.97 (1.90-4.63) | <0.001 | 36.8% |
| Asians | 2 (325) | 0.73 (0.54-0.97) | 0.020 | 0% | 3.39 (2.11-5.44) | <0.001 | 0% |
| **BRPC** |  |  |  |  |  |  |  |
| Matched patient factors^a^ | 4 (185) | 0.52 (0.34-0.80) | 0.003 | 0% | 5.70 (2.49-13.04) | <0.001 | 29.7% |
| Matched tumor size | 4 (258) | 0.71 (0.51-1.00) | 0.047 | 0% | 3.62 (1.91-6.85) | <0.001 | 22.1% |
| Matched vascular resection | 4 (258) | 0.71 (0.51-1.00) | 0.047 | 0% | 3.62 (1.91-6.85) | <0.001 | 22.1% |
| Matched initial CA19-9 level | 4 (185) | 0.52 (0.34-0.80) | 0.003 | 0% | 5.70 (2.49-13.04) | <0.001 | 29.7% |
| Matched tumor factors^b^ | 3 (115) | 0.57 (0.31-1.04) | 0.066 | 0% | 4.79 (1.94-11.84) | 0.001 | 43% |
| Matched patient and tumor factors | 3 (115) | 0.57 (0.31-1.04) | 0.066 | 0% | 4.79 (1.94-11.84) | 0.001 | 43% |
| Pancreatic head cancer (≥80% of patients) | 1 (51) | 0.47 (0.19-1.14) | 0.094 | NA | 2.62 (0.82-8.34) | 0.104 | NA |
| Matched adjuvant therapy | 5 (353) | 0.71 (0.53-0.94) | 0.015 | 0% | 2.44 (1.46-4.09) | 0.001 | 40.5% |
| Asians | 5 (416) | 0.66 (0.51-0.85) | 0.001 | 0% | 2.32 (1.38-3.90) | 0.001 | 10% |

| Abbreviation: RPC, resectable pancreatic cancer; BRPC, borderline resectable pancreatic cancer; NAC(R)T+R, neoadjuvant chemo(radio)therapy followed by resection; HRs, hazard rates; ORs, odds ratios.  Note: HRs below 1 favor NAC(R)T+R, whereas ORs over 1 favor NAC(R)T+R.  ^a^Patient factors including age and sex. | ^b^Tumor factors including initial tumor size and location, vascular resection and initial CA19-9 level.  ^c^This sensitivity analysis included Tajima et al because of the study contained a subset of pancreatic head cancer that was available. |
| --- | --- |

**Table S8.** **Quantitative Assessment for Asymmetry of Funnel Plots.**

|  | **Overall survival**  **in all patients** | | | **Overall survival**  **in resected patients** | | |  |
| --- | --- | --- | --- | --- | --- | --- | --- |
|  | **RPC+BRPC** | **RPC** | **BRPC** | **RPC+BRPC** | **RPC** | **BRPC** |  |
| **Number of studies** | | 11 | 6 | 4 | 15 | 6 | 6 |
| **P value for Begg’s tests** | | 0.482 | 1.00 | 0.308 | 0.023 | 0.060 | 0.260 |
| **P value for Egger’s tests** | | 0.148 | 0.840 | 0.064 | 0.018 | 0.147 | 0.443 |

**Figure S1. Funnel plots of Overall Survival in all the patients (A-C) and resected patients (D-G).
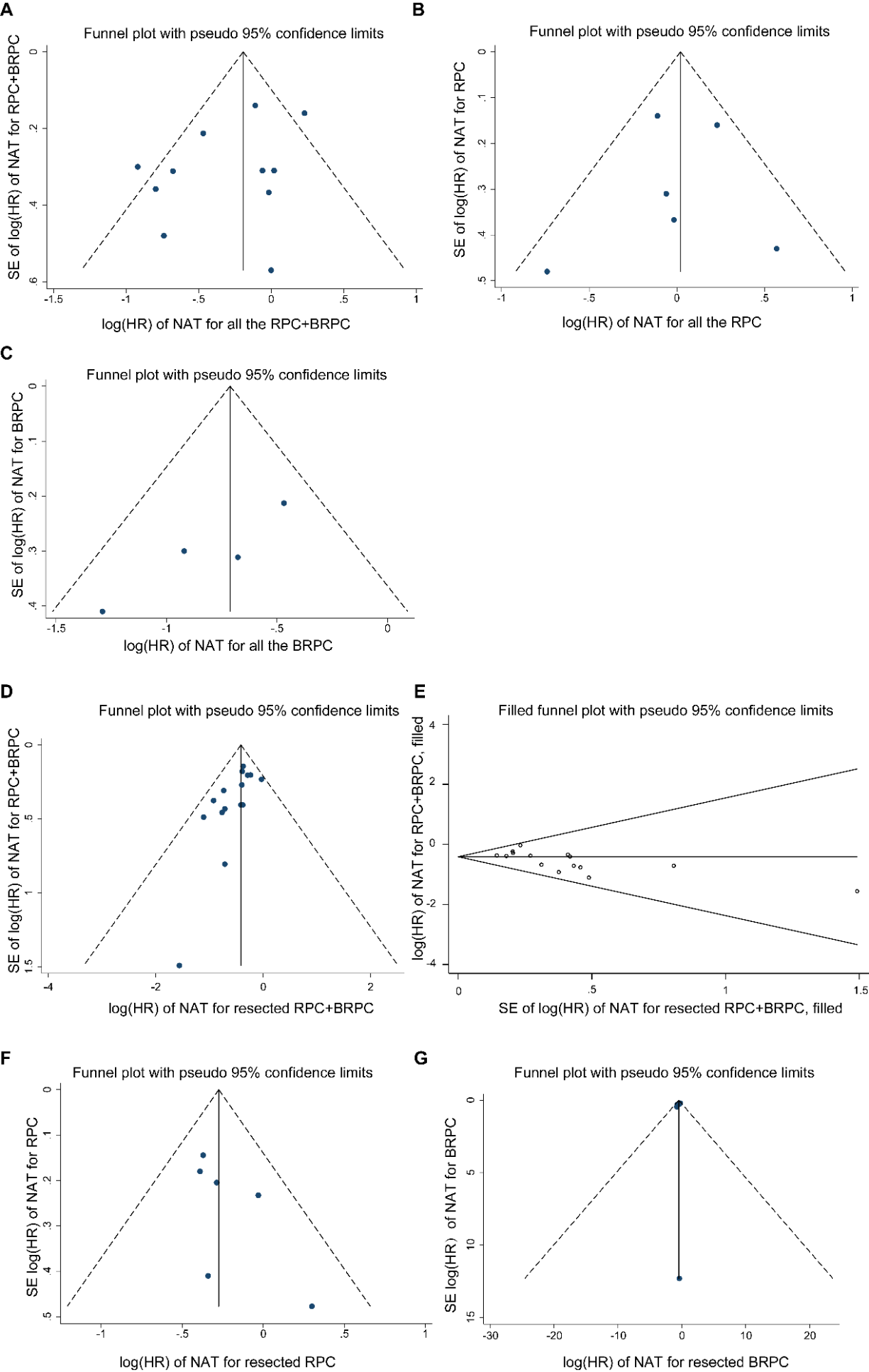
**
